# Molecular Mechanisms of the Methionine Sulfoxide Reductase System from *Neisseria meningitidis*

**DOI:** 10.3390/antiox7100131

**Published:** 2018-10-01

**Authors:** Sandrine Boschi-Muller

**Affiliations:** Ingénierie Moléculaire et Physiopathologie Articulaire (IMoPA), UMR 7365 CNRS-Université de Lorraine, Bâtiment Biopole, Faculté de Médecine, 54506 Vandoeuvre-lès-Nancy, France; sandrine.boschi@univ-lorraine.fr; Tel.: +33-372-746-662

**Keywords:** methionine sulfoxide, MSR, PilB, Trx, periplasm, disulfide-bond formation (Dsb) D

## Abstract

*Neisseria meningitidis*, an obligate pathogenic bacterium in humans, has acquired different defense mechanisms to detect and fight the oxidative stress generated by the host’s defense during infection. A notable example of such a mechanism is the PilB reducing system, which repairs oxidatively-damaged methionine residues. This review will focus on the catalytic mechanism of the two methionine sulfoxide reductase (MSR) domains of PilB, which represent model enzymes for catalysis of the reduction of a sulfoxide function by thiols through sulfenic acid chemistry. The mechanism of recycling of these MSR domains by various “Trx-like” disulfide oxidoreductases will also be discussed.

## 1. Introduction

*Neisseria meningitidis* is the infectious agent responsible for meningitis and septicemia. Its pathogenicity depends on its ability to resist activated oxygen and nitrate species produced by host macrophages in response to infection. For this, *N. meningitidis* has developed multiple antioxidant defense systems, including the PilB reducing system, which is capable of repairing the sulfoxide form of oxidatively-damaged methionine residues.

The *pilB* gene was initially identified in *Neisseria gonorrhoeae* in 1988, and its name derives from early studies that suggested that PilB was a transcriptional regulator inhibiting pilin synthesis [1,2,3]. However, this hypothesis was discredited in 2002 when PilB was shown not to play any role in pilin expression. Indeed, the transformation of this strain with a plasmid allowing the production of PilB under the control of an isopropylthiogalactoside (IPTG)-dependent promoter, had no effect on piliation [4].

**Figure 1:**
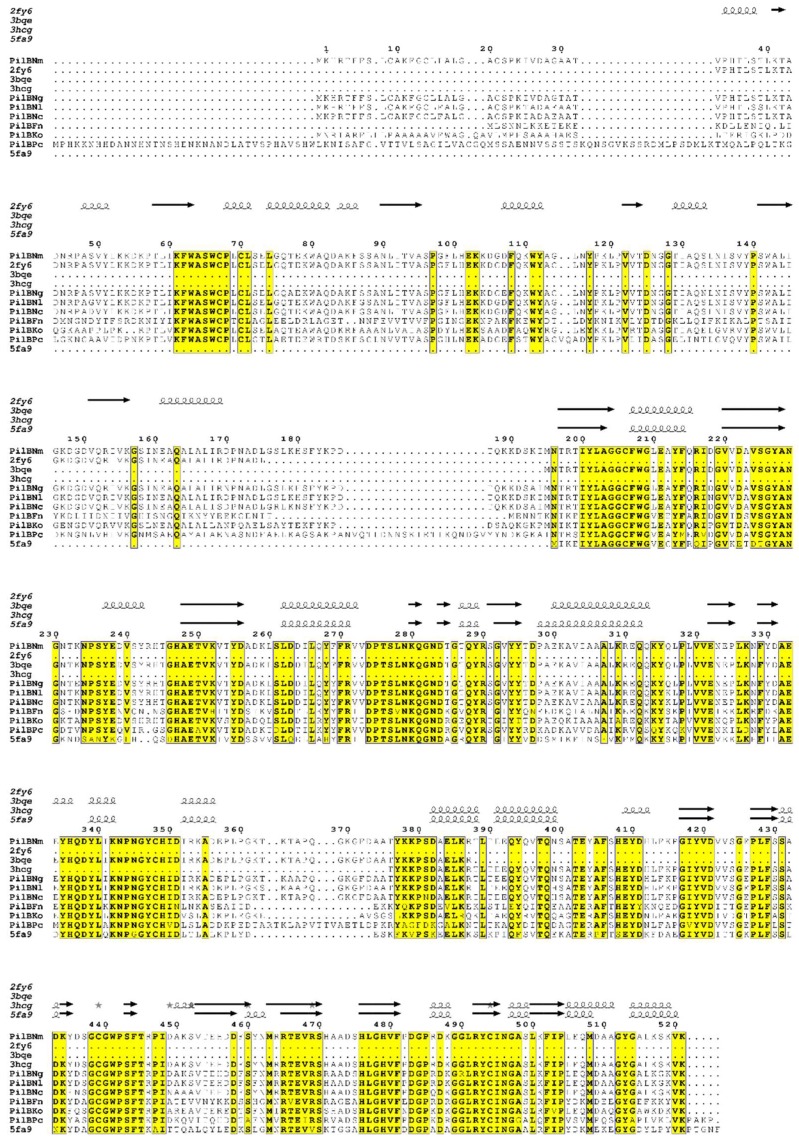
Multiple-sequence alignment of PilB homologues in *N. meningitidis* (PilBNm), *N. gonorrhoeae* (PilBNg), *N. lactamica* (PilBNl), *N. cinerae* (PilBNc), *Fusobacterium nucleatum* (PilBFn), *Kingella oralis* (PilBKo) and *Psychrobacter cryohalolentis* (PilBPc) and secondary structures of *N. meningitidis N*-ter (2fy6), MSRA (3bqe) and MSRB (3hcg) domains. MSRAB fusion of *Treponema denticola* (5fa9) is also presented. The amino acid numbering is based on *N. meningitidis* PilB. Conserved residues at 80% are indicated in black on yellow boxes. Stars indicate residues with alternate conformations. The linker regions between *N*-ter and MSRA, and MSRA and MsrB are located between residues 176 and 195, and 357 and 382, respectively. The alignment was carried out using ClustalW [6], and the figure was prepared using ESPript (http://espript.ibcp.fr [7]). MSR: methionine sulfoxide reductase.

Analysis by protein sequence identity of publicly-available translated genome data, shows that the PilB protein is encoded by only a few bacterial genomes. These include human commensal and pathogenic bacteria such as *Neisseriae* (*N. meningitidis*, *N. gonorrhoeae*, *N. lactamica* and *N. cinerea*), as well as the bacteria present in dental plaque, such as *Fusobacterium nucleatum* and *Kingella oralis*, and the non-pathogenic extremophile bacterium *Psychrobacter cryohalolentis* (Figure 1). The moderate to high sequence conservation (sequence identities between 47 and 98%) and the environmental proximity of some of these bacteria, suggest that the gene was acquired by horizontal transfer between species, conferring the capacity to fight oxidative stress.

**Figure 2:**
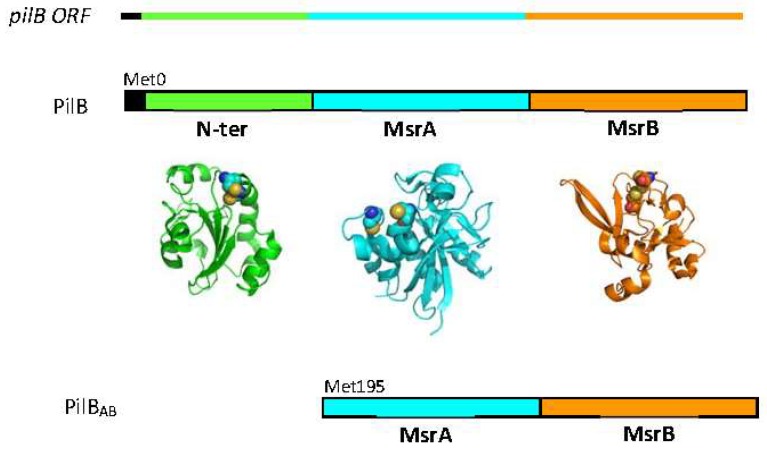
The *N. meningitidis pilB* ORF contains a signal peptide (black) and the coding sequences for the three functional domains (green for *N*-ter, cyan for MSRA and orange for MSRB). The three domain protein (PilB) is located in the periplasm, whereas the cytoplasmic form contains only the two MSR domains (PILB_AB_). The structures of the isolated *N*-ter (pdb 2fy6), MSRA (pdb 3bqe) and MSRB (pdb 3hcg) domains were generated using the PyMol program (DeLano Scientific LLC, San Carlos, CA, USA).

PilB is composed of three domains: a thioredoxine (Trx)-like *N*-terminal domain (*N-*ter) with disulfide oxidoreductase activity, and two domains (named A and B) with methionine sulfoxide reductase (MSR) activity. In *Neisseriae*, two protein forms derived from the *pilB* gene have been characterized: a truncated cytoplasmic form called PilB_AB_ containing only the two MSR domains and a periplasmic form called PilB, incorporating the three domains [4] (Figure 2). From heterologous expression studies in *Escherichia coli*, the truncated form of *N. meningitidis* PilB has been shown to be produced by an internal reinitiation mechanism during translation, at an AUG codon corresponding to the Met195 residue of the whole protein [5].

Studies by the Seifert group have shown that the PilB protein of *N. gonorrhoeae*, which is probably located on the outer membrane within the periplasm, is involved in the resistance to oxidative stress, as it is necessary for the survival of bacteria in the presence of H_2_O_2_ [4]. This activity is in all likelihood conferred by its MSR domains, as has been demonstrated for MSR enzymes from other bacteria [8]. However, the PilB system exhibits several peculiarities relative to these discrete homologues: the location of MSRs in both the cytoplasm and the periplasm, the fusion of the MSRA and MSRB activities into a single protein, and the presence of an essential *N*-ter domain. The MSR-dependent repair mechanisms of *N. meningitidis* PilB have been extensively studied in vitro and in this review, we summarize the molecular mechanism of sulfoxide reduction by MSR domains, the role of the *N*-ter in the periplasm, and information concerning the periplasmic recycling partners.

## 2. Methionine Sulfoxide Reductase Activities of PilB

### 2.1. MSRs of N. meningitidis

MSRs catalyze the reduction of methionine sulfoxide (Met-O) to methionine (Met) in the presence of reducing agent. To date, three structurally distinct classes of MSRs have been characterized: MSRA, MSRB and fRMSR. MSRA, MSRB and fRMSR are cysteine-containing enzymes which display different substrate specificities: MSRA and MSRB specifically reduce the *S*- and *R*-isomers of free or peptide-bound *L*-Met-O, respectively, whereas fRMSR (for “free-R-MSR”) only reduces the *R*-isomer of free Met-O, with high selectivity for *L*-Met-*R*-O [9,10,11]. The oxidation of methionine residues produces both diatereoisomers of *L*-Met-O, referred to as *R* and *S*, due to the presence of a second asymmetric center on the sulfur atom of the sulfoxide function. Thus, the presence of both MSRA and B activities is necessary to allow for full reduction of *L*-Met-*R*, *S*-O back to *L*-Met.

In *Neisseriae*, PilB_AB_ and PilB carry both MSRA and B functions, while an isolated cytoplasmic protein exhibits an fRMSR activity, the role of which is not known [12]. The chemical and catalytic mechanism of the MSR domains of PilB as well as their three-dimensional structures have been extensively studied, as they represent model enzymes for classical two cysteine MSRs, as discussed in previous reviews [13,14,15,16]. The results are thus only summarized briefly here.

### 2.2. Catalytic Mechanism of MSR Domains

**Figure 3:**
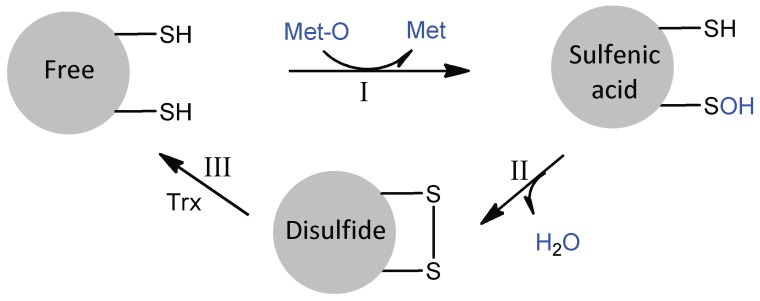
The three-step mechanism of MSRs. In step I, the formation of the MSR/Met-O Michaelis complex allows the nucleophilic attack of catalytic Cys on the sulfur atom of the sulfoxide function of Met-O, leading to the formation of a sulfenic acid intermediate with concomitant release of Met. In step II, the nucleophilic attack of the regenerating Cys on the sulfur atom of the sulfenic acid leads to the formation of a disulfide bond and the release of a water molecule. In step III, the regeneration of the active site in its fully reduced state proceeds via the reduction of the MSR disulfide bond by reduced Trx. For *N. meningitidis* MSRA and MSRB, the catalytic and regenerating Cys are C51 and C198, and C117 and C63, respectively.

The PilB MSRA and MSRB domains are typical two-cysteine MSRs, which act by a common three-step mechanism involving sulfenic acid chemistry (Figure 3) [17,18]. In the absence of a reducing agent, the reduction of Met-O leads to the successive formation of a sulfenic acid intermediate on the catalytic cysteine residue and a disulfide between the catalytic and regenerating cysteine residues, concomitant to the release of a water molecule. The rate of the first step has been shown to be very fast for both enzymes (790 s^−1^ for MSRA, 85 s^−1^ for MSRB, with Ac-L-Met-*R*-O-NHMe as a model substrate), such that the rate-limiting step of the reaction in the absence of reducing agent is disulfide bond formation. This observation shows that although MSRs A and B have clearly distinct 3D structures [19,20,21], their actives sites are adapted to efficiently catalyze the reduction of a sulfoxide by a thiol, suggesting a convergent evolution.

**Figure 4:**
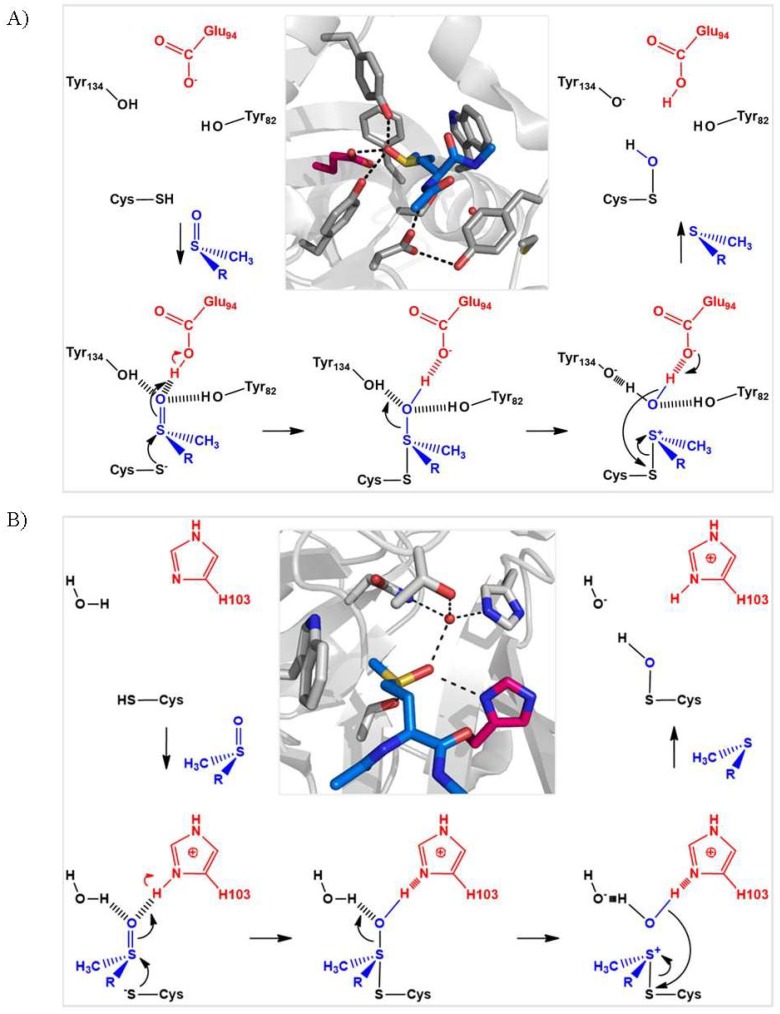
Proposed scenario for formation of the sulfenic acid intermediate in MSRA (**A**) and MSRB (**B**). In the Michaelis complex, the oxygen of the sulfoxide function of Met-O (in blue) is stabilized by hydrogen-bonding interactions. The transfer of a proton from the acid catalyst (in red) leads to the formation of a sulfurane, followed by that of a sulfonium cation and a water molecule via protonation. The activated water molecule then attacks the Cys sulfur of the sulfonium cation to generate the sulfenic acid and Met. For *N. meningitidis* MSRA and MSRB, the catalytic cysteine, general acid catalyst and proton donor are Cys51, the carboxylate of Glu94 and Tyr82 or Tyr134, and Cys 117, the imidazolium ring of His103 and a water molecule, respectively. The representations of the active sites of C51S MSRA (pdb 3bqf) and C117S MSRB (pdb 3hch) in complex with Ac-*L*-Met-*S*-ONHMe and Ac-*L*-Met-*R*-ONHMe, respectively, were generated using the PyMol program (DeLano Scientific LLC).

In terms of catalysis, the mechanism involves the deprotonation of the catalytic Cys and protonation of the oxygen atom of the sulfoxide function of the substrate, in order to facilitate the nucleophilic attack of the thiolate and the formation of a sulfurane-type transition state (Figure 4). These proton transfers are achieved via the participation of a general acid base catalyst bearing a carboxylate or imidazole functional group for MSRA and MSRB, respectively. It is generally accepted that the formation of the sulfurane is rate-determining, and that this species evolves into a sulfonium cation which is ultimately attacked by an activated water molecule to produce the sulfenic acid intermediate, although definitive experimental data to support this hypothesis are still lacking [15,22,23,24]. Overall, this catalytic strategy depends on the strong polarization of the sulfoxide function, which facilitates both the transfer of the oxygen atom and the reduction of the sulfoxide function.

In the presence of the reducing agent, the regeneration of the reduced form of MSRs is achieved through the reduction of the disulfide bond by a disulfide oxidoreductase exhibiting a Trx fold. Indeed, the cytoplasmic PilB_AB_ use Trx, whereas this role is played by the *N*-ter in the periplasm (see Section 3). For isolated MSRA or MSRB enzymes, reduction by Trx is the rate-limiting step of the overall mechanism. For both disulfide oxidized MSRA and MSRB, this recycling process involves the initial formation of a productive oxidized MSR/reduced Trx complex, followed by the two electron-transfer chemical step leading to the accumulation of the reduced MSR/oxidized Trx complex, whose dissociation is overall rate-limiting [13,25,26]. This mechanism implies that specific structural recognition should occur between oxidized MSRs and reduced Trx, which is likely a prerequisite for the efficiency of the oxidoreduction process. Although the structure of a covalent PilB_AB_-Trx complex has not been solved to date, the inter-partner interface is likely to involve the limited but highly complementary hydrophobic surfaces typical of redox partners that exhibit short-lived interactions [27].

### 2.3. Substrate Specificities of MSR Domains

The numerous kinetic and structural studies carried out on MSRs domains have not yet permitted the identification of all the molecular factors underlying their substrate specificities. Nonetheless, it is clear that the presence of the sulfoxide function is a prerequisite for binding to both MSRs. The active sites of MSRA and MSRB are both located on the surface of the enzymes and contain two mirror-image sub-sites which confer stereospecificity for the *R* or *S* stereoisomer of L-Met-O: (1) a hydrophilic pocket involving the stabilization of the protonated sulfoxide function of the substrate via hydrogen bonding; and (2) a hydrophobic pocket consisting essentially of the side chain of a Trp residue, which stabilizes the ε-methyl group of the substrate via its indole ring (Figure 4) [9,13,28,29]. The selectivity of the enzymes for protein-bound Met-O versus free Met-O probably derives from a destabilizing effect, due to repulsive interactions with the charged amino and/or carboxyl groups of the free amino acid.

### 2.4. Catalytic Efficiency of MSRA/MSRB Fusion

The fusion of MSRA and MSRB domains is not specific to PilB. Indeed, fusion proteins are found in more than 46 bacteria including *Treponema denticola, Streptococcus pneumoniae* and *Shewanella oneidensis*, for example, but the functional interest of such fusions remains poorly understood [30,31]. As mentioned earlier, due to their complementary stereospecificity, the presence of both MSR activities allows the reduction and thus repair of both forms of oxidized Mets in proteins. The question rather thus concerns the kinetic efficiency of the fusion protein compared to isolated enzymes.

The catalytic efficiency (*k*_cat_/*K*_M_) of PilB_AB_ under steady-state conditions was in fact found to be similar to that of the discrete enzymes in the presence of their preferred substrates. Thus, the fusion does not significantly alter the catalytic efficiency of the MSRs, suggesting that the two function as independent domains even within the context of PilB [18]. This contrasts with kinetic studies of other MSRA/MSRB fusion proteins, which revealed an increase in catalytic efficiency relative to the discrete enzymes (for example in *S. oneidensis* and *T. denticola* [30,31]). In the latter case, the higher catalytic efficiency of the fusion protein derives from hydrogen bond interactions between MSRA and MSRB and the linker lying between the two domains. Interestingly, this linker loop is about 13 residues longer in PilB, suggesting that the precise way in which the MSRs are fused may differ between species (Figure 1).

## 3. Recycling of MSR Activities within PilB

### 3.1. The N-ter Domain

In order for the catalytic activity to be regenerated, MSRs need to be reduced at the end of the catalytic cycle, a function performed by Trx in the cytoplasm and the *N*-ter domain in the periplasm. The *N*-ter domain of PilB is a disulfide oxidoreductase of the “Trx-like” family, with a TlpA fold and a WCPLC disulfide center redox potential of −0.230 V. Isolated *N*-ter domain was shown to reduce both isolated MSRA and MSRB domains [5], although it exhibits higher catalytic efficiency towards the MSRB domain (second-order constant *k*_2_ approximately 10^4^ M^−1^.s^−1^). In this later case, the dissociation of the reduced MSRB/oxidized *N*-ter complex remains rate-limiting, as in the case of Trx. For MSRA (*k*_2_ approximately 10^2^ M^−1^.s^−1^), the formation of the oxidized MSRA/reduced *N*-ter complex occurs more slowly and is in fact rate-limiting, probably reflecting the existence of a structural fit between the oxidized MSRA domain and the reduced *N*-ter domain (data not shown). Although the structure of the Nter/MSR complexes are not known, the interactions seem to be limited to the active site area as described for other complexes involving “Trx-like” oxidoreductases.

X-ray diffraction and NMR studies of the *N*-ter domain revealed the presence of a rigid ^99^FLHE^102^ loop covering one edge of the active site, in both its oxidized and reduced states [32,33]. This additional loop, which is not found in Trxs or in other TlpAs, plays a critical role in recognizing the PilB MSRs. Indeed, the catalytic efficiency (*k*_2_ values) for the reduction of the MSR domains by a truncated form of the *N*-ter, in which the loop has been genetically removed, is 10-fold less efficient relative to the intact *N*-ter [34].

### 3.2. Recycling of MSRs Activities within PilB

**Figure 5:**
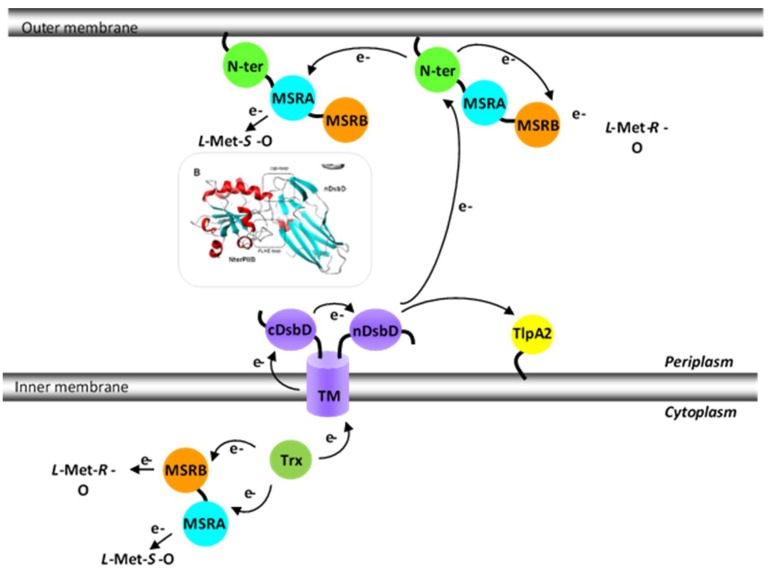
*N. meningitidis* Met-O reducing pathways. The electrons used by the MSR domains of PilB originate from the cytoplasmic Trx and are furnished either directly to the domains (in the case of the cytoplasmic PilB_AB_) or via the intermediary of the three domains of disulfide-bond formation (Dsb) D (transmembrane TM, cDsbD and finally nDsbD) and the *N*-ter domain (in the case of periplasmic PilB). The *N*-ter/nDsdD model is also represented.

In PilB, the *N*-ter domain efficiently reduces only the MSRB domain via an intramolecular mechanism, whereas the reduction of the MSRA domain depends on the classical Trx-like intermolecular mechanism (data not shown) (Figure 5). The formation of an intramolecular disulfide bond between the *N*-ter and MSRB domains, which is kinetically favored compared to the intermolecular reaction, implies either a spatial proximity of the MSRB and *N*-ter domains in PilB or that the presence of long "linker" regions between the two domains allows sufficient flexibility for the formation of this transient disulfide bond. Examination of the PilB sequences reveals the existence of linkers between the three functional domains. Specifically, a linker of 20 amino acids is found between the *N*-ter and MSRA domains, and one of 26 amino acids between the MSRA and MSRB domains, in *N. meningitidis* PilB (Figure 1). The presence of these long linkers, the second of which is approximately 13 residues longer than that in the MSRA-MSRB fusion of *T. denticola* [31], suggests that PilB should show overall greater flexibility, which allows the *N*-ter and MSRB domains to approximate each other, and which additionally explains the observed independence of the MSR domains in kinetic studies. Obtaining a three-dimensional structure of PilB would, however, provide additional valuable information on the spatial organization of the three domains with respect to each other.

In conclusion, the in vitro results suggest that the mechanisms for reducing the MSRA and MSRB domains in PilB are different: the reduction of MSRB occurs intramolecularly, while MSRA is regenerated intermolecularly. As to the situation in vivo, PilB is a periplasmic protein that deploys its MSR activities to reduce the Met residues of oxidized periplasmic targets. In vivo, the reductive regeneration of the two MSR domains must therefore be efficient in the periplasm to allow the bacterium to cope with oxidative stress. Based on in vitro data, however, the reduction of MSRA is expected to be slower relative to MSRB. As the MSRB domain is specific for the reduction of Met-*R*-O, but the oxidation of Met leads to an equimolar mixture of *R* and *S* isomers, PilB should only be able to efficiently regenerate the half of the Met that is oxidized. Thus, either this activity is sufficient for the bacterium to transiently resist oxidative stress, or the reduction of the *S* isomer, although quite slow, is sufficient to restore the function of oxidized targets. A final possibility is that reduction of the MSRA domain involves another periplasmic “Trx-like” protein, opening up the possibility to efficiently regenerate both the Met-O stereoisomers.

## 4. Periplasmic Recycling Partners

To efficiently reduce oxidized Met, PilB has to itself be reduced in the periplasm. This role is fulfilled by the disulfide-bond formation (Dsb) D protein, the general transmembrane redox hub protein that uses the reducing power of Trx transferred to periplasmic “Trx-like” disulfide oxidoreductases via specific interactions with its *N*-terminal domain, called nDsbD [35] (Figure 5). The *N. meningitidis* nDsbD is able to reduce periplasmic “Trx-like” such as the *N*-ter domain [34]. The catalytic efficiency of this reduction step is high, with *k*_2_ values of 6 × 10^5^ M^−1^ s^−1^, suggesting the existence of specific recognition between nDsbD and *N*-ter. The formation of the nDsbD-*N*-ter complex requires the opening of the nDsbD “cap-loop” region which is positioned above the *N*-ter redox center. This conformational adaptation would be a prerequisite for the formation of a catalytically-competent complex leading to the formation of an intermolecular disulfide bond between nDsbD and the “Trx-like” partner. It should be noted that *N*-ter FLHE loop does not seem to be essential for the formation and stabilization of the complex.

nDsbD efficiently reduces the *N*-ter domain of PilB in vitro, but since the DsbD protein is a three domain transmembrane protein localized in the inner membrane, the PilB protein must be located nearby to be able to interact with the nDsbD in vivo. However, studies by Skaar and colleagues in 2002 showed using a subcellular fractionation approach that *N. gonorrhoeae* PilB is anchored to the outer membrane, which is incompatible with the use of DsbD as a reducing partner [4]. However, as the outer membrane anchoring of PilB appears to be weak (it involves hydrophobic interactions between the membrane and a small amphipathic *N*-terminal helix (amino acids 4–22) [5]), the alternative localization of PilB, such as in the interspace where it may be associated with peptidoglycan or near the internal membrane, cannot be excluded.

## 5. Conclusions

During the past decades, the MSR system of *N. meningitidis* was a model study of choice in many respects. The MSR domains of PilB, which have been the subject of extensive investigation at both the kinetic and structural levels, represent model enzymes for catalysis of the reduction of a sulfoxide function by thiols through sulfenic acid chemistry. The PilB protein is able to reduce Met-O in the cytoplasm but also in the periplasm, a compartment particularly subject to oxidation due to the permeability of the outer membrane, by a unique molecular mechanism combining the catalytic advantages of modular proteins, conformational flexibility and a Trx-like recognition mode.

The contribution of PilB to the virulence of *N. meningitidis* suggested that it might be a good therapeutic target, in particular for the treatment of serogroup B infection for which no vaccine is available. However, the similarities between bacterial and host MSR enzymes could limit the success of this strategy. In this context, bacterial specific periplasmic Trx-like proteins, such as the *N*-ter of PilB or TlpA2 (involved in the essential cytochrome c maturation process [36]), may be more promising targets.
